# Assuring the Biofunctionalization of Silicone Covalently Bonded to Rhamnolipids: Antibiofilm Activity and Biocompatibility

**DOI:** 10.3390/pharmaceutics14091836

**Published:** 2022-08-31

**Authors:** Maïssa Dardouri, Ana Bettencourt, Victor Martin, Filomena A. Carvalho, Bruno Colaço, Adelina Gama, Madeleine Ramstedt, Nuno C. Santos, Maria H. Fernandes, Pedro S. Gomes, Isabel A. C. Ribeiro

**Affiliations:** 1 Research Institute for Medicines (iMed.ULisboa), Faculty of Pharmacy, Universidade de Lisboa, Avenida Prof. Gama Pinto, 1649-003 Lisboa, Portugal; 2 BoneLab—Laboratory for Bone Metabolism and Regeneration, Faculty of Dental Medicine, University of Porto, Rua Dr. Manuel Pereira da Silva, 4200-393 Porto, Portugal; 3 LAQV/REQUIMTE, University of Porto, Praça Coronel Pacheco, 4050-453 Porto, Portugal; 4 Instituto de Medicina Molecular, Faculdade de Medicina, Universidade de Lisboa, Av. Prof. Egas Moniz, 1649-028 Lisbon, Portugal; 5 Animal and Veterinary Research Centre (CECAV), Associate Laboratory for Animal and Veterinary Science–AL4AnimalS, University of Trás-os-Montes and Alto Douro (UTAD), 5000-801 Vila Real, Portugal; 6 Department of Chemistry, Umeå University, 901 87 Umeå, Sweden

**Keywords:** rhamnolipids, PDMS, flow chamber, translational rabbit model, antimicrobial, biocompatible

## Abstract

Silicone-based medical devices composed of polydimethylsiloxane (PDMS) are widely used all over the human body (e.g., urinary stents and catheters, central venous catheters stents) with extreme clinical success. Nevertheless, their abiotic surfaces, being prone to microorganism colonization, are often involved in infection occurrence. Improving PDMS antimicrobial properties by surface functionalization with biosurfactants to prevent related infections has been the goal of different works, but studies that mimic the clinical use of these novel surfaces are missing. This work aims at the biofunctional assessment of PDMS functionalized with rhamnolipids (RLs), using translational tests that more closely mimic the clinical microenvironment. Rhamnolipids were covalently bonded to PDMS, and the obtained surfaces were characterized by contact angle modification assessment, ATR-FTIR analysis and atomic force microscopy imaging. Moreover, a parallel flow chamber was used to assess the *Staphylococcus aureus* antibiofilm activity of the obtained surfaces under dynamic conditions, and an in vitro characterization with human dermal fibroblast cells in both direct and indirect characterization assays, along with an in vivo subcutaneous implantation assay in the translational rabbit model, was performed. A 1.2 log reduction in *S. aureus* biofilm was observed after 24 h under flow dynamic conditions. Additionally, functionalized PDMS lessened cell adhesion upon direct contact, while supporting a cytocompatible profile, within an indirect assay. The adequacy of the biological response was further validated upon in vivo subcutaneous tissue implantation. An important step was taken towards biofunctional assessment of RLs-functionalized PDMS, reinforcing their suitability for medical device usage and infection prevention.

## 1. Introduction

Medical devices made of silicone (polydimethylsiloxane, PDMS) are widely used in various applications in the body (e.g., urinary stents and catheters, central venous catheters stents) with high clinical success [1,2,3,4]. However, silicone surfaces are prone to favour microorganism adhesion which may evolve into device-associated biofilm development. These infections are very difficult to manage with antibiotics, considerably limiting the lifetime of the device and are associated with increased healthcare costs and patient’s morbidity [5,6]. Specifically, current therapeutic approaches using antibiotics are hampered by reduced penetration of the drugs through biofilm and low accumulation levels at infected sites, requiring prolonged usage [7,8]. Presently, the search for antimicrobial agents is focused among other approaches, on the use of natural compounds that limit the toxicity of conventional drugs and provide a potential solution to the antimicrobial resistance crisis [9,10]. In this context, the functionalization of the silicone surface with natural antimicrobial compounds may be a potential alternative to the pristine material.

**Figure 1:**
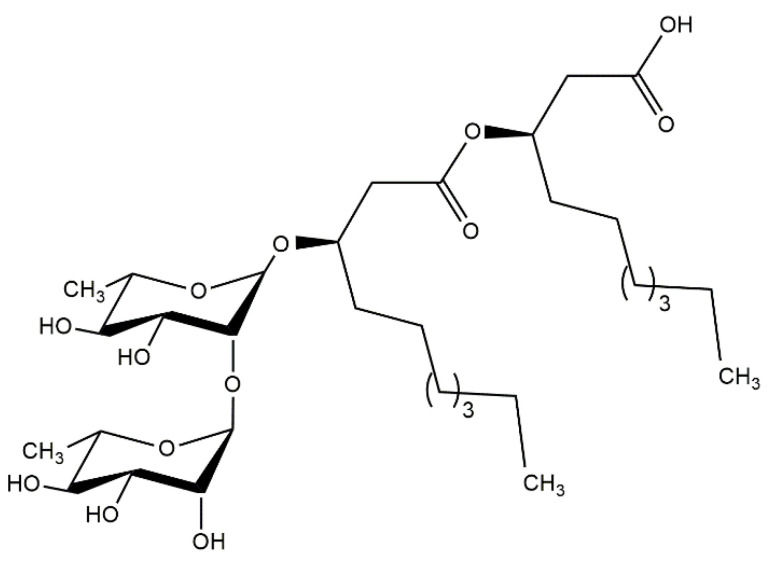
Main RL present in RLs mixture produced by *Pseudomonas aeruginosa*, here designated as di-RL RhaRhaC10:C10:0.

Previous studies have shown that among natural compounds with antimicrobial properties are rhamnolipids (RLs). RLs are biosurfactants mainly biosynthesized by *Pseudomonas aeruginosa* and *Burkholderia* strains. They occur as mixtures and are composed of one or two rhamnose molecules linked to one, two or sometimes three β-hydroxyalkanoic acids (Figure 1) [11]. These biosurfactants when covalently bonded to the PDMS surface lead to a reduction in microorganism (*Staphylococcus aureus*, *Staphylococcus aureus* and *Candida albicans*) colonization as assessed by antimicrobial assays under static conditions [12,13]. Moreover, PDMS–RLs surfaces have also been tested for in vitro cytotoxicity (with human fibroblastic cells) and hemocompatibility (regarding platelet adhesion), as well as in vivo irritation potential (hen’s egg-chorioallantoic membrane test, HET-CAM) [13].

Since the first evaluation level of the biological evaluation of PDMS functionalized with RLs showed promising results, the present work aims at a further step in the evaluation of its potential biomedical interest. Specifically, the second evaluation level was focused on a biofunctional assessment using translational tests that more closely mimic the clinical microenvironment. In this context, antibiofilm properties of RL-functionalized PDMS surfaces were assessed under flow conditions on a parallel flow chamber. When working under dynamic conditions (i.e., under flow), antimicrobial compounds and their interaction with microbial cell may be influenced. The adhesion of bacterial cells is also affected and consequently biofilm formation and structure [14]. Moreover, when considering contact killing/antiadhesive surfaces submitted to flow conditions, material surfaces may be covered by microbial debris, which can reduce antimicrobial activity [14].

Diverse biocompatibility testing approaches were conducted using a thorough in vitro characterization with human dermal fibroblast cells in both direct and indirect characterization assays, along with an in vivo subcutaneous implantation assay in the translational rabbit model.

Finally, to confirm the adequacy of RLs production and silicone functionalization, a range of complementary techniques was used, namely ultra-high performance liquid chromatography coupled to tandem mass spectrometry (UHPLC–MS/MS), attenuated total reflection Fourier-transform infrared (ATR-FTIR), contact angle and atomic force microscopy (AFM) analysis.

## 2. Materials and Methods

### 2.1. Chemicals, Solvents and Materials

The following chemicals and solvents were used: sulfuric acid 98%, methanol, glacial acetic acid, MES buffer, sodium hydroxide, potassium dihydrogen phosphate and 4-methoxy benzaldehyde(p-anisaldehyde from Merck (Darmstadt, Germany); chloroform and ethanol (100%) from Carlo Erba (Val-de-Reuil, France); N-hydroxysuccinimide (NHS), N-(3-dimethylaminopropyl)-N′-ethyl carbodiimide hydrochloride (EDC), D-(+)-glucose monohydrate and (3-aminopropyl) triethoxysilane (APTES) from Sigma-Aldrich (Saint Louis, MO, USA); sodium chloride from Panreac (Barcelona, Spain); acetonitrile and hydrochloric acid 37% from Scharlau (Sentmenat, Spain); hydrogen peroxide 30% from VWR Chemicals (Leuven, Belgium); peptone, agar and yeast extract from Biokar Diagnostics (Beauvais, France); deionized water obtained from a MilliQ system.

### 2.2. Rhamnolipids Characterization

Rhamnolipids (90% purity), produced by *P. aeruginosa,* obtained from Sigma (AGAE Technologies, Corvallis, OR, USA) were characterized by UHPLC–MS/MS according to a previously described method [15].

### 2.3. PDMS Surface Functionalization

#### Chemical Etching and RLs Functionalization

FDA-approved medical grade PDMS (SOVE, Queluz, Portugal) with 0.5 mm thickness, was cut into 1 × 1 cm squared pieces and functionalized. In brief, the PDMS surface was activated by oxidation with a “piranha solution”, APTES was bonded to the PDMS surface, and RLs were linked to APTES through the carbodiimide reaction according to Dardouri et al. [13]. After being washed 3 times with Type I (Milli-Q) water, samples were dried under vacuum and stored until further use.

### 2.4. Surface Characterization

#### 2.4.1. Wettability

The wettability of the PDMS surfaces was evaluated using the contact angle sessile drop technique. MilliQ water droplets (2 µL) were placed on top of each surface, and images were acquired (Rohs USB Digital Microscope, China). Contact angles were measured using the Image J software (1.52p, National Institutes of Health, Bethesda, MD, USA). Measurements were performed 10 min after the droplet deposition and results expressed as the average of at least 8 independent measurements.

#### 2.4.2. Fourier-Transform Infrared Spectroscopy (FTIR)

Chemical functional groups present in samples surfaces were assessed by attenuated total reflectance Fourier transform infrared spectroscopy (ATR-FTIR, Bruker Alpha II). Samples were placed on the ATR diamond crystal, and the spectra measurements with a resolution of 4 cm^−1^ ranged between 4000 and 800 cm^−1^. The acquisition of data and their treatment were performed with the software OPUS 7.8.

#### 2.4.3. AFM

The roughness of the functionalized/non-functionalized PDMS surfaces was analysed by atomic force microscopy (AFM). A NanoWizard IV atomic force microscope (JPK Instruments, Berlin, Germany) mounted on the top of an Axiovert 200 inverted optical microscope (Carl Zeiss, Jena, Germany) was used for scanning images of surfaces. The AFM head was equipped with a 15 μm z-range linearized piezoelectric scanner and an infrared laser. Imaging of the PDMS surfaces was performed in air, in contact mode. Oxidized sharpened silicon pyramidal tips with a tip radius of approximately 6 nm, resonant frequency of about 190 kHz and spring constant of 58 N/m were used for the imaging. Imaging parameters were adjusted to minimize the force applied on the scanning of the topography of the PDMS surfaces. Scanning speed was optimized to 0.3 Hz, and acquisition points were 512 × 512. Imaging data were analysed with the JPK image processing v.6.0.55 (JPK Instruments, Berlin, Germany).

### 2.5. Antibiofilm Activity under Dynamic Conditions

#### 2.5.1. Microorganisms

Frozen stoked (−80 °C) *S. aureus* ATCC 25923^TM^ from the American Type Culture Collection (ATCC) cultured on tryptic soy agar (TSA, Biokar Diagnostics, Beauvais, France) plates for 24 h at 37 °C were used for the antibiofilm activity assessment.

#### 2.5.2. Flow Cell Assay

Antibiofilm activity under dynamic conditions was accessed with a flow system model BST FC270 (Biosurface Technology Corporation, Bozeman, MT, USA) coupled to a peristaltic pump (Gilson, MINIPLUS 3) programmed for a flow rate of 1.2 mL/min, according to [16]. Samples of pristine PDMS and PDMS–RLs were placed in triplicate in the flow chamber compartment, and at least two independent experiments were performed. RPMI 1640 (1X) GlutaMAX^TM^ medium (GIBCO) was 10-fold diluted in sterile PBS, added of sodium pyruvate (100 μM_,_ Merck, Darmstadt, Germany), uracil (9 μM, Merck, Darmstadt, Germany) and sodium nitrite (100 μM, Merck, Darmstadt, Germany) according to [17] and used as experimental medium. Inoculum was prepared from a 24 h culture of *S. aureus* and further diluted in experimental medium until reaching 10^6^ CFU mL^−1^. Final inoculum was injected in each channel of the flow chamber, and *S. aureus* cells were allowed to adhere to the surface of the material for 30 min without flow, after which the flow was resumed. After 24 h, *S. aureus* biofilm formed at the material surface was assessed by CFU counts according to [13].

### 2.6. Biocompatibility

**Figure 2:**
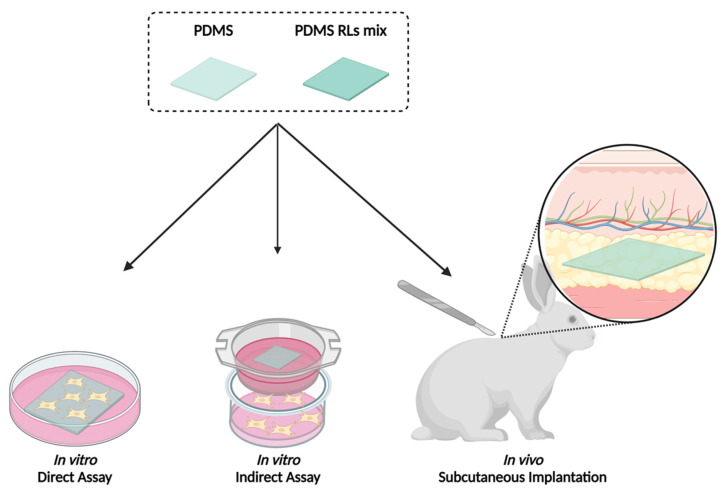
Schematics of the biocompatibility assessment of PDMS materials, conjoining in vitro and in vivo techniques.

The biocompatibility of the PDMS materials was evaluated through three different approaches, two in vitro cytocompatibility assessment techniques (focusing on the cellular response to the PDMS materials’ surfaces—direct assay; and the cell response to their leachates—indirect assay), and the in vivo response to the materials’ subcutaneous implantation, as shown in Figure 2.

#### 2.6.1. In Vitro Biocompatibility Assessment

In vitro biocompatibility assays were performed using human dermal fibroblasts cultures (HDFs, AG22719, Coriell Institute for Medical Research, Camden, NJ, USA) from the 4th passage. Briefly, HDFs were grown in α-minimal essential medium (α-MEM) supplemented with fetal bovine serum (FBS) 10% *v*/*v*, 100 IU/mL penicillin, 100 μg/mL streptomycin and 2.5 μg/mL amphotericin B (all from Gibco, Gaithersburg, MD, USA) and incubated at 37 °C and 5% CO_2_ in air. After reaching approximately 70% confluence, cells were trypsinized and sub-cultured at 1 × 10^4^ cells/cm^2^ in two distinct experimental approaches: (a) direct contact, in which cells were seeded directly over the PDMS materials to evaluate the direct interaction between cells and surfaces and (b) indirect contact, in which cells were seeded in the bottom of the culture plate with a permeable insert (Transwell 6.5 mm insert, 0.4 µm permeable membrane, Costar, Tewksbury, MA, USA) fitted with the PDMS materials, allowing the passage of their leachates to the culture medium and the evaluation of the cellular function. Cells cultured without materials were used as control, while materials incubated without cells were used as blank.

##### Cell Viability/Metabolic Activity of the Culture

Cell viability/metabolic activity of the cultures was evaluated using the resazurin assay. At determined time points, culture medium was replaced by fresh medium containing 10% *v*/*v* of resazurin solution (Sigma-Aldrich, Saint Louis, MO, USA) at 100 µg/mL, following a 3 h incubation at 37 °C. After the incubation period, media were collected, and a microplate reader (Synergy HT; BioTek, Winooski, VT, USA) was used to measure samples’ fluorescence at 530/590 nm (excitation/emission wavelengths). Experiments were performed in quintuplicate.

##### Cell Morphology

HDFs morphology was observed by fluorescence imaging throughout filamentous actin (F-actin) staining using phalloidin-conjugated Alexa Fluor 488 (Molecular Probes, Eugene, OR, USA) and nucleus counterstaining using Hoechst 33342 (Enzo, Farmingdale, NY, USA). 24 h cell cultures from direct and indirect assays were fixed with paraformaldehyde 3.7%, permeabilized with 0.1% Triton-X (Sigma-Aldrich) and incubated with bovine serum albumin (Sigma–Aldrich) prior to staining. Images were obtained using a Celena S digital imaging system (Logos Biosystems, Annandale, VA, USA) and were processed using the ImageJ software v.1.53k (National Institutes of Health, Bethesda, MD, USA).

#### 2.6.2. In Vivo Biocompatibility Assessment

A total of 12 male New Zealand White rabbits, 17 weeks old and weighing 3500–3700 g were acquired from a certified vendor, acclimatized, housed and maintained as previously described by the research team [18]. Procedures and descriptions were performed in compliance with the ARRIVE guidelines.

Briefly, animals were sedated with an intramuscular injection of midazolam (1 mg/kg) and anesthetized upon the administration of ketamine (25 mg/kg) and xylazine (5 mg/Kg). Through the procedure, sterile saline was continuously administered at 10mL/kg/h, and animals were maintained on a heated surface. Upon the confirmation of the anesthetic plane, animals were tricotomized in the dorsal region, and the skin was disinfected with a chlorhexidine solution. Cutaneous incisions were performed, and materials were randomly distributed upon blunt subcutaneous dissection and hemostatic control. The surgical wounds were then closed with Polyglactin 910-4/0 restorable suture, with single stitches. An analgesic protocol was established and continued for 3 days with buprenorphine (0.03 mg/kg). For each animal, 6 material samples were randomly implanted (3 PDMS and 3 PDMS-RLs). At each of the defined time points—2, 7 and 28 days—animals (*n* = 4) were euthanized with an anesthetic overdose. Tissue samples were harvested, fixed in formalin and prepared for histological characterization through hematoxylin and eosin (H&E) staining to evaluate tissue response and Masson’s trichrome staining to evaluate capsule formation. Sections were photo documented with an Olympus BX-51/22 dotSlide digital virtual microscope.

### 2.7. Statistical Analysis

In AFM analysis and dynamic antibiofilm assay, the statistical analysis was performed by applying the unpaired samples Student’s t test. GraphPad Prism 5 software v.5.03 (GraphPad Software, San Diego, CA, USA) was used to perform the statistical analysis.

In biocompatibility assays the Shapiro–Wilk test was used to evaluate the normality of the attained data. Regarding normal data sets, one-way ANOVA was performed, followed by Tukey’s test for multiple comparisons. For non-parametric data sets, the Kruskal–Wallis test was performed, followed by Dunn’s tests for multiple comparisons; *p* values ≤ 0.05 were considered significant.

In all assays, quantitative data were presented as mean ± standard error.

## 3. Results and Discussion

### 3.1. RLs Mixture Characterization

Before PDMS surface functionalization, the RLs mixture was characterized by UHPLC-MS analysis. Mono and Di-RLs were identified. It was possible to conclude that identified RLs presented different fatty acid in their composition with different unsaturated stages. The major RL congener was RhaRhaC10:0C10:0 representing 35% of the total mixture. Other RLs present in the mixture are described in Table 1. Although a different batch of RLs mixture was used in PDMS functionalization in previous works [13], it was possible to observe that the qualitative composition of mixtures was similar (Table 1).

### 3.2. Functionalized Samples Characterization

To assure surface functionalization, contact angle was measured, ATR-FTIR spectra of surfaces was performed and AFM imaging of surfaces was obtained. The covalent bonding of RLs onto the surface increased the surface wettability since a water contact angle obtained after 10 min was 27 ± 4° for PDMS– RLs, whereas 85 ± 10° was obtained for pristine PDMS (Table 1). This decrease in the wettability will influence the adhesion of *S. aureus* bacteria, which prefer hydrophobic environments, as previously observed [6].

ATR-FTIR spectra allowed the identification of RLs representative bands present in -PDMS-RLs samples, namely: symmetrical and asymmetrical vibration of CH groups at 2960 and 2883 cm^−1^ and C=O stretching from ester at 1740 cm^−1^ [12,19] (Table 1). Moreover, characteristic peaks of the amide functional group appeared, namely, the N-H bond at 1565 cm^−1^ and the C=O bond at 1668 cm^−1^_,_ indicating the successfully covalent binding of RLs onto PDMS [20].

Finally, the bonds from PDMS were also visible at: 2965 cm^−1^, representative of C-H stretching vibrations of -CH_3_; 1263 cm^−1^_,_ due to the various vibration of the C-H bonds from the PDMS methyl groups Si-(CH_3_)_2_ and 1014 and 1087 cm^−1^, related to the stretching vibration of Si-O-Si bonds.

Additionally, when comparing with previously functionalized samples obtained from a different batch of RLs mixture [13], wettability results and ATR-FTIR peaks of functionalized samples were similar among different batches (Table 1).

**Figure 3:**
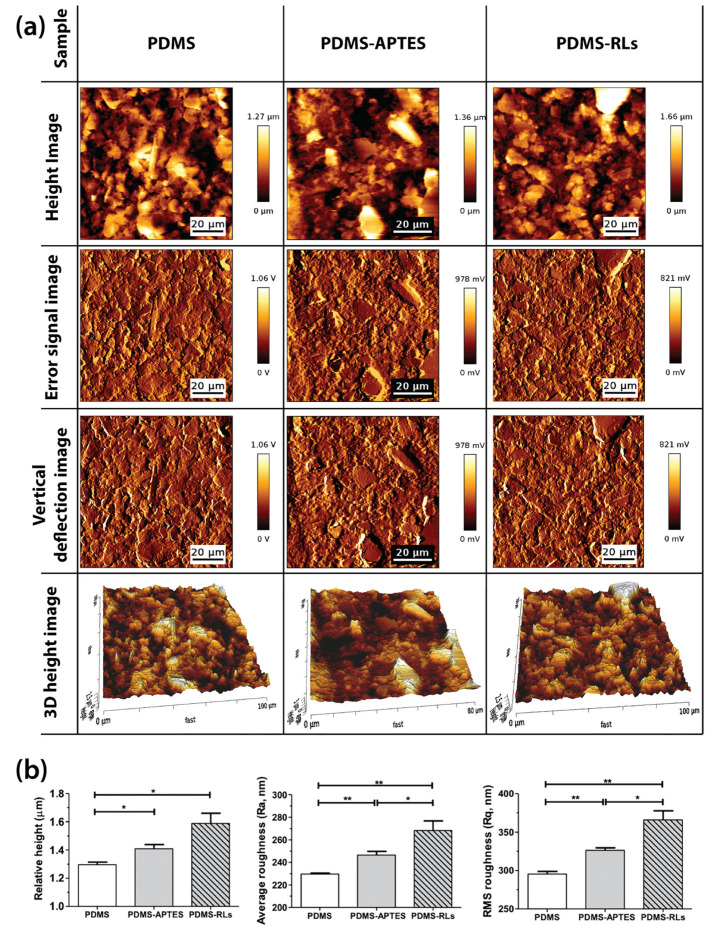
(**a**) Atomic force microscopy images of PDMS, PDMS–APTES and PDMS–RLs. Height AFM images at a non-fixed scale (1st column). Error signal and vertical deflection signal images (2nd and 3rd column, respectively). 4th column: AFM height “3D image” at a fixed scaled of 1 µm and error signal image (100 µm × 100 µm); (**b**) relative height, average roughness (Ra) and root mean square (RMS) roughness (Rq) data obtained from the AFM images of PDMS, PDMS–APTES and PDMS–RLs surfaces (relative height: * *p* < 0.029; Ra: * *p* = 0.045; ** *p* < 0.0073; Rq: * *p* = 0.027; ** *p* < 0.0057).

AFM analysis (Figure 3) shows the three-dimensional AFM scanning height images, where it is possible to compare the differences between the pristine PDMS, functionalization with APTES (only) and with RLs. Oxidation and silanization with APTES slightly increased PDMS surface height and the roughness. In contrast, the RLs addition to the PDMS surface significantly increased the height and the roughness of the surface. These results suggest a surface restructuring with the binding of the compounds.

### 3.3. Antibiofilm Activity under Dynamic Conditions

**Figure 4:**
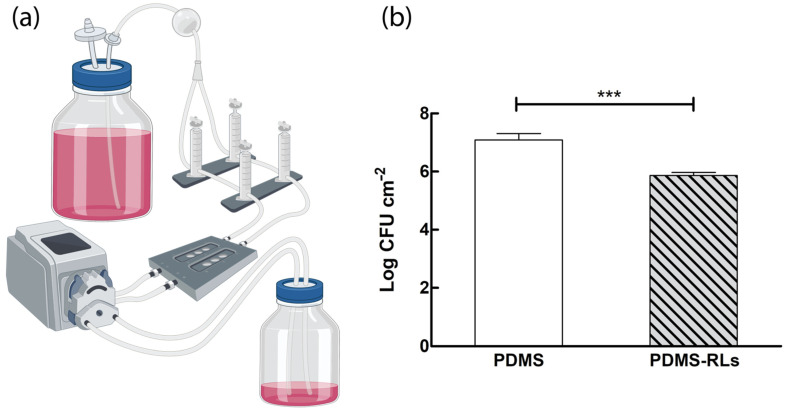
(**a**) Schematic representation of the flow dynamic assay to characterize surfaces antibiofilm activity; (**b**) *S. aureus* biofilm inhibition by RLs-functionalized surfaces under dynamic conditions assessed by colony forming unit counts (*** *p* < 0.01).

To assess the ability of the herein suggested RLs-coating in inhibiting biofilm formation under dynamic conditions, CFU counts were performed on samples submitted to those assays (Figure 4a). Regarding the antibiofilm activity of RLs-functionalized PDMS, it was possible to observe a 1.2 log (93.4%) reduction of *S. aureus* when compared to pristine PDMS (Figure 4b).

In recent works, some antibiofilm assays have been developed on PDMS functionalized with RLs, either by adsorption or covalent bond, under static conditions with the intention of mimicking medical devices surfaces, such as those of catheters [12,13,21]. Under static conditions, covalent functionalization of PDMS with RLs has shown activity towards methicillin sensitive and resistant *S. aureus*, *S. epidermidis* [12] and *S. aureus*, *S. epidermidis* and *C. albicans* when in mono and dual culture biofilms [13]. Nevertheless, when introduced in the human body, catheters are subjected to dynamic conditions. Thus, assays that intend to simulate in some way the use of the catheter are of added value to elucidate the suitability of the proposed antibiofilm approach. The in vitro dynamic model described here enabled us to emulate catheter usage and biofilm formation on its surface. Others have already mentioned the advantages of similar experimental methodologies to identify effective agents in the treatment of catheter-related infections. For example, Hogan et al. evaluated the activity of several common antibiotics, antiseptics [22] and even newer antimicrobial agents (ML:8 and Citrox) [23] against *S. aureus* biofilms formed and treated within a flow cell model. In our study, the model allowed us to elucidate that even under contact dynamic conditions, the covalent functionalization of PDMS with RLs was able to reduce *S. aureus* biofilm formation.

### 3.4. Biocompatibility

#### 3.4.1. In Vitro Biological Evaluation

**Figure 5:**
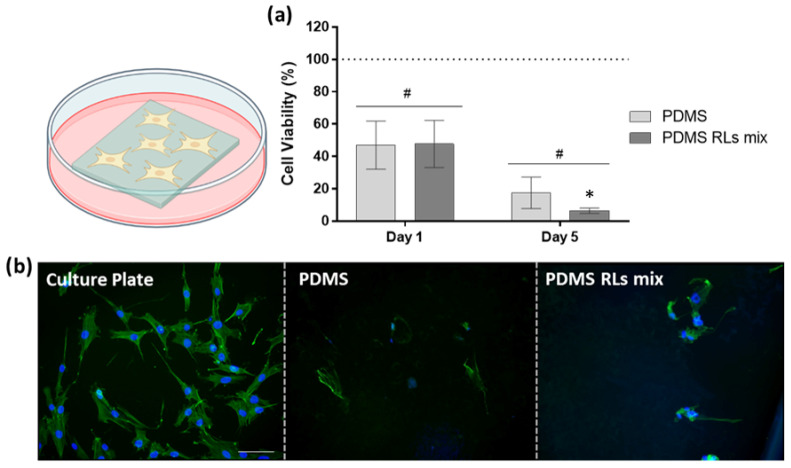
Direct assay—HDFs cultures were seeded directly over PDMS’ materials: (**a**) cell viability/metabolic activity assessed by resazurin assay; cells seeded on the culture plate were used as control, set as 100%; # significantly different from control; * significantly different between experimental conditions, *p* ≤ 0.05; (**b**) representative fluorescence images of the cell morphology and culture organization at 24 h; actin cytoskeleton was stained in green, while the nuclei were counterstained in blue; scale bar: 100 µm.

The biological response of the fibroblastic cell cultures varied significantly depending on the experimental setting—with the direct evaluation aiming to disclose the cell functionality in direct contact with the PDMS-based materials, and the indirect evaluation focusing on disclosing the cytocompatibility of the materials within a biological environment. As in the direct assay, HDF had difficulty to adhere and/or proliferate over PDMS materials, as demonstrated by the MTT assay (Figure 5a). At day 1, cell viability was significantly reduced (between 25% and 50%) in comparison to control, while at day 5, the viability’s reduction was even more pronounced. Among experimental groups, PDMS–RLs presented the most intense reduction, reaching around a 90% decrease at day 5. These findings were corroborated by cell morphology imaging (Figure 5b)—in control cultures, HDFs were found to be stretched, occupying a high area of the culture plate with orderly arranged actin fibers distributed through the cytoplasm. Furthermore, intercellular contacts were noted by the presence of filopodia and other cytoplasmic extensions. In contrast, cells were scarce over PDMS materials, and an impaired morphology was noted in addition to cellular debris and remnants.

Within the indirect assays, cell viability/metabolic activity was found to be merely reduced (around 10–15%) at day 1 in experimental PDMS materials in comparison to control. In contrast, at day 5, all experimental groups (PDMS materials) presented a higher cell viability/metabolic activity than control, with the PDMS–RLs group attaining significance higher levels. Moreover, no differences in cell morphology were noted in comparison to control, suggesting a biocompatible profile within the indirect assay.

**Figure 6:**
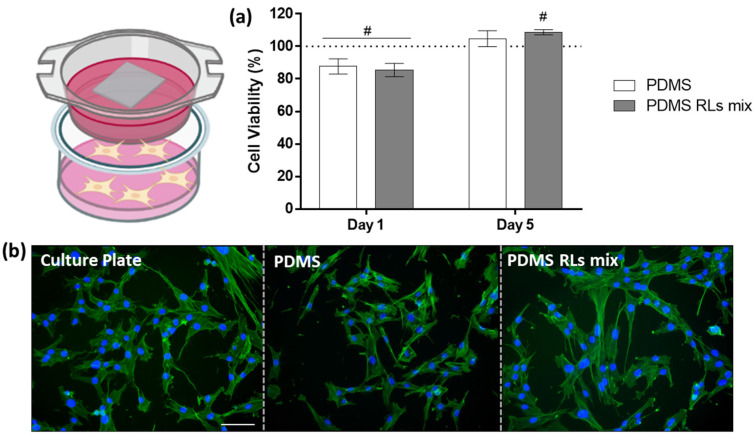
HDF cultures were seeded on the bottom of culture plates and incubated indirectly in the presence of PDMS materials: (**a**) cell viability/metabolic activity assessed by resazurin assay; cells seeded over the culture plate were used as control, set as 100%; # significantly different from control; *p* ≤ 0.05; (**b**) representative fluorescence images of cell morphology of 24 h HDF cultures; actin cytoskeleton was stained in green while the nuclei were counterstained in blue; scale bar: 100 µm.

In the present study, PDMS-modified materials displayed cell-repellent characteristics demonstrated by the direct assessment (Figure 5) and simultaneously, a suitable cytocompatibility to human fibroblastic cells grown in the same microenvironment, as demonstrated within the indirect approach (Figure 6). The PDMS–RLs group presented optimal results transversely within both assays, attaining the lowest cell viability/metabolic activity in the direct assay, while its leachate reached higher values in relation to plain PDMS. The noted decrease in cell adhesion and overall cell viability over the materials’ surface can be attributed to PDMS physic-chemical characteristics, as the non-polar nature [24] may lessen the adsorption of selected proteins, inducing a low ligand-mediated cell spreading and preventing cell adhesion and maturation, ultimately compromising cell functionality [25]. Our findings lined up with other studies in the literature—Kidambi et al. reported that fibroblasts struggle to adhere on plain PDMS surfaces, presenting a proliferation rate close to zero [26]. Moreover, alternative PDMS functionalization set on the addition of substances such as dopamine, heparin, trypsin and topography modifications [26,27,28] achieved similar results. Furthermore, oxidization of the PDMS surface can avoid fibronectin adsorption, preventing a successive cell adhesion [29]. Overall, RLs functionalization of the PDMS surface was found to be highly effective in precluding adhesion and proliferation of cellular elements, essential for the targeted clinical application.

Finally, the incubation with a material’s leachates is important to ensure that non-toxic substances were released towards not only the cellular microenvironment, but also within the vascular system, potentially hampering the cellular and tissue functionality. Our data demonstrated the cytocompatibility of PDMS leachates, from either non-functionalized and RLs-functionalized surfaces, substantiating the cytocompatible profile of the developed materials. This is in accordance with the results of Bordenave et al., which demonstrated PDMS leachable cytocompatibility with endothelial cell populations [30].

#### 3.4.2. In Vivo Biological Evaluation

Within the postoperative period, no complications were identified until euthanasia, with animals presenting an adequate recovery and behavioral response as well as an adequate physiological response regarding gastrointestinal and urinary functionality. Normal clinical signs were also verified regarding cardiac and respiratory function, body temperature and mucosal coloration. After euthanasia, at the postmortem evaluation, no significant alterations were identified on major organs, and no macroscopic tissue alterations were verified within the implantation region. In addition, no significant alterations (such as degeneration, necrosis, excessive inflammatory infiltrate or fibrosis were observed in the histopathological analysis.

**Figure 7:**
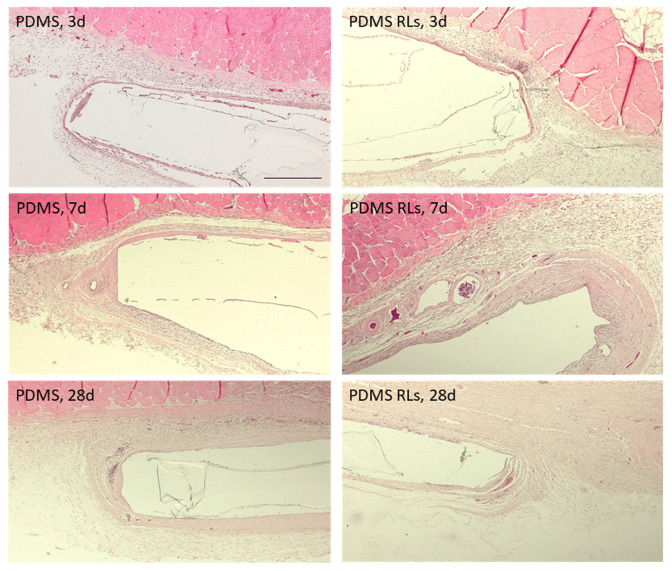
Representative photomicrographs of the histological tissue sections following PDMS and PDMS–RLs implantation, at 3, 7 and 28 days, stained with hematoxylin and eosin. Scale bar: 150 µm.

**Figure 8:**
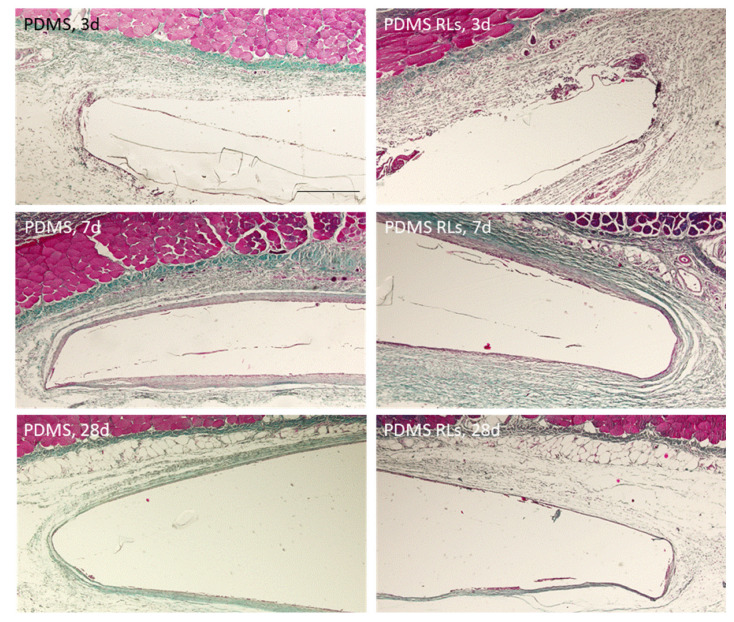
Representative photomicrographs of the histological tissue sections following PDMS and PDMS–RLs implantation, at 3, 7 and 28 days, stained with Masson’s trichrome. Scale bar: 150 µm.

The tissue response was characterized for up to 28 days, revealing the formation of a thin fibrous connective tissue capsule around the implanted PDMS membranes as well as the development of neovascular structures and inflammatory cellular infiltration. Comparatively, differences were found regarding the characterized periods and implanted materials, with a trend for an increased tissue reaction verified at earlier time points and within the PDMS–RLs formulation. At days 2 and 7, tissue adjacent to the implantation area presented fibrin and the accumulation of inflammatory populations, constituted mainly of neutrophils and scattered eosinophils (Figure 7). Simultaneously, a thin collagenous capsule could be identified, particularly evident with the Masson trichrome staining (Figure 8). At later implantation time points, 4 weeks, a significant reduced inflammatory infiltrate was attained for both implanted formulations, with a population shifting to the predominance of macrophages and lymphocytes (Figure 7 and Figure 8).

The biological response attained upon implantation is consistent with previous findings in which the immunological activation follows an acknowledged timeline as a result of the foreign material implantation and the response to the surgical injury [31]. Characteristically, the initial polymorphonuclear infiltrate is progressively substituted by the recruitment of lymphocytes and macrophages and commonly associated with a collagenous encapsulation, broadly avascular in the most severe forms [32]. In the present analysis, for both PDMS and PDMS–RLs, a mild response was verified, with a moderate cellular infiltration that diminished progressively into the later time points, substantiating a biocompatible outcome and integration with neighboring tissues. Additionally, only a thin collagenous deposition was verified around the assayed materials in association with a significant amount of newly formed blood vessels, which is in line with a favorable outcome [33]. In fact, the organization of a dense collagenous/fiber capsule around implants impedes not only the proper interaction with the tissues, but also further impairs gas, nutrients and waste products exchange, thus compromising their functionality [33]. In accordance with the present results, PDMS-based materials have previously been reported to have an excellent biocompatibility, being extremely stable, thus keeping their flexibility—essential for the required applications, and presenting with a high resistance to biodegradation, assuring functionality upon implantation with minor evidence of foreign body reaction [34].

## 4. Conclusions

This work aimed at the biofunctional evaluation of RLs-functionalized PDMS by the use of translational tests that mimic a clinical microenvironment. Antibiofilm activity of obtained antimicrobial surfaces was assessed in a parallel flow chamber, and a reduction of 1.2 log units was observed after 24 h under dynamic flow conditions. Cytocompatibility studies revealed a decreased cellular adhesion and the absence of a cytotoxic behavior in direct and indirect assays, respectively. In vivo subcutaneous implantation assays further substantiated the adequacy of the tissue response.

With this work, an important step was taken towards the proof of concept for the further use of RLs-functionalized PDMS able to prevent medical device-related infections.

## Figures and Tables

**Table 1:** Characterization of RLs mixture and functionalized PDMS surfaces used in this work and compared with a previous publication [13]. RLs mixture used in the present and previous work were obtained from 2 different batches (Sigma Aldrich), designated here as B_1_ and B_2,_ respectively. RLs Mixture (B2) data was adapted from [13].

Assay	Designation	RLs Mixture (B_1_)	RLs Mixture (B_2_)
**UHPLC-MS**	RhaRhaC10:0C8:0	+	+
	RhaC8:0C10:0	+	+
	RhaRhaC8:0C12:0	+	+
	RhaRhaC10:0C10:1	+	+
	RhaRhaC10:C12:1	+	+
	RhaRhaC10:0C12:0	+	+
	RhaC10:0C12:1	+	+
	RhaC10:0C10:1	+	+
	RhaRhaC10:0C14:1	+	+
	RhaC10:0C12:0	+	+
**Contact angle** **(°)**	**PDMS**	85 ± 10	95 ± 4
	**PDMS-RLs**	27 ± 4	17 ± 2
**ATR-FTIR (cm^−1^)**	**RLs mixture**		
	CH symmetrical stretch	2960 *	2960 *
	CH asymmetrical stretch	2883 *	2883 *
	C=O from ester */carboxylic	1740 *	1740 *
	CH2 deformation	1408	1408
	C-O-C in the rhamnose ring	1078	1145
	**PDMS**		
	C-H stretch of CH_3_	2965 *	2965 *
	C-H of Si-(CH_3_)_2_	1263 *	1263 *
	Si-O-Si stretch	1014 * and 1087 *	1014 * and 1087 *
	**PDMS-RLs**		
	C=O bond of the amide	1668	1650
	N-H bond of the amide	1565	1565
	C-N bond of the amide	-	1450
	Other bands present marked with *		

Note: Specific RLs identified in each batch were marked as +; Specific funtional groups of RLs and PDMS also detected in PDMS-RLs samples were marked with * in RLs mixture and PDMS rows.
